# Chronic Cold-Water-Induced Hypothermia Impairs Memory Retrieval and Nepeta menthoides as a Traditional “Hot” Herb Reverses the Impairment 

**Published:** 2014

**Authors:** Mohammad Mahdi Ahmadian-Attar, Abolhassan Ahmadiani, Mohammad Kamalinejad, Leila Dargahi, Mahmoud Mosaddegh

**Affiliations:** a *Department of Traditional Pharmacy, School of Traditional Medicine, Shahid Beheshti University of Medical Sciences,Tehran, Iran.*; b *Neuroscience Research Center, Shahid Beheshti University of Medical Sciences, Tehran, Iran. *; c *Department of Pharmacology, Faculty of Medicine, University of Malaya, 50603, Kuala Lumpur, Malaysia.*; d *Department of Pharmacognosy, School of Pharmacy, Shahid Beheshti University of Medical Sciences, Tehran, Iran. *

**Keywords:** Brain cold intemperament, Alzheimer’s disease, *Nepeta menthoides*, Learning and memory, Tau hyperphosphorylation

## Abstract

Iranian Traditional Medicine (ITM) describes a kind of dementia with similar signs and symptoms of Alzheimer’s disease (AD). It explains the pathology of dementia with cold intemperament of the brain, which means that the brain is colder than its healthy form. ITM strategy for treatment of dementia is to heat the brain up by medical “hot” herbs. *Nepeta menthoides *(NM) is one of these “hot” herbs. To evaluate the veracity of ITM concept about dementia and its treatment, we first try to examine if coldness of brain can make memory impairment. If so, can NM reverse memory impairment? Rats in cold-water-induced hypothermic (CWH) groups were immersed up to the neck in 3.5 °C water, for 5 min during 14 consecutive days. As a control, rats were forced to swim in warm water at the same conditions. To eliminate the impact of forced swimming stress, a group of intact rats was also added. After last swimming in day 14, some groups received drug (100 or 500 mg/ Kg aqueous extract of NM) or vehicle via *i.p*. injection. Learning and memory were assessed by Morris water maze, and tau hyperphosphorylation was measured by western blotting. The results showed that CWH impairs learning and memory and induces tau hyperphosphorylation. 100 mg/Kg of NM reversed memory impairment as well as tau hyperphosphorylation. ITM theory about the relationship between brain hypothermia and dementia is in accordance with our findings.

## Introduction

Iranian traditional medicine (ITM) had been used among peoples who lived beyond the official borders of what we now know as Iran for quite a long time. The far-reaching history of its use among different people with different races and cultures makes this very medical school full of ethnopharmacological experiences, including the treatment of dementia. ITM describes a kind of dementia with similar signs and symptoms of Alzheimer’s disease (1). It explains the pathology of this kind of dementia with cold intemperament of the brain, which means that the brain is colder than its regular, healthy form (2, 3). ITM strategy for treatment of this kind of dementia is to heat the brain up by either physical heat or medical “hot” herbs (2, 3). *Nepeta menthoides *Boiss. (called Ostokhoddus in Persian) is one of these “hot” herbs (4-6).

The genus *Nepeta *is one of the common genuses in Iran flora which is ethnopharmacologically prescribed for respiratory, gastric, and nervous ailments (7, 8). Among the members of this genus, *Nepeta menthoides *(Labiatae) is traditionally used for some psychological and cerebral disorders like anxiety and nervous disorders (8). In ITM, it is believed that a syrup of this herb consisted of water extract of aerial flowering parts of the herb and honey can ameliorate forgetfulness by heating the brain (6). ITM has also warns about overdose of this medicine; because it can make the brain hotter than its natural balance and cause forgetfulness by itself.

To evaluate the veracity of ITM concept about dementia and its treatment, we first try to examine if coldness of brain can make memory impairment. If so, can *Nepeta menthoides *as a “hot” herb reverse memory impairment? In this article, we try to answer these questions by the aid of animal behavioral analysis as well as hippocampal molecular investigations.

## Experimental

*Animals*

Adult male Wistar rats (200–230 g) were obtained from our own breeding colony. They were housed in groups of six animals per cages with free access to food and water. They were maintained on a 12 h-light/dark cycle (light on at 07:00), at a temperature of 23 ± 1 °C. All experiments were carried out according to the Guide for the Care and Use of Laboratory Animals published by the US National Institute of Health (NIH Publications No. 8523, revised in 1985). All efforts were made to minimize animal suffering and to reduce the number of animals used.

*Plant extraction*

*Nepeta menthoides *(aerial part) was obtained from Tehran botanical market and authenticated in the Herbarium of Faculty of Traditional Medicine, Shahid Beheshti University of Medical Sciences in comparison with original samples. According to the policy of the herbarium, no specific number is given for such a sampling but the sample is kept for occasional checking

during the study. Water extract of the herb was prepared by infusion and was concentrated in a vacuum rotary evaporator (Buchi, Switzerland) and was left to dry in a desiccator. The yield of extraction (w/w) was 23% which was calculated as weight of dry extract/weight of dry starting materialˣ100. The extract was diluted at desirable concentrations in normal saline prior to injections.

*Cold-water-induced hypothermia*

According to two studies on the impact of cold-water swimming on brain temperature (9, 10), we utilized 3.5 °C cold-water to induce hypothermia. Rats in cold-water-induced hypothermic (CWH) groups were immersed up to the neck in 3.5 ± 0.5 °C water of 20 cm depth in a 35 cm diameter plastic container, for 5 min during 14 consecutive days. After each intervention, rats were left to dry at room temperature 23 ± 2 °C and returned to the cages. For control groups,

rats were forced to swim in warm water (32 °C) at the same conditions. After last swimming in day 14, some groups received drug or vehicle (details are presented in Table 1). To eliminate the impact of forced swimming stress, a group of intact rats was also added. All procedures were carried out on day time. 24 hours after last intervention, spatial learning and memory was assessed using Morris water maze test.

**Table 1:** Description of animal groups

**Group name**	**Intervention**
CWH (Hypothermic), n=17	cold-water-swimming for 14 consecutive day
Ctr (Control), n=17	warm-water swimming for 14 consecutive day
CWH + 100, n=8	cold-water-swimming for 14 consecutive day plus single i.p. injection of *Nepeta menthoides *(100 mg/kg)
CWH + 500, n=6	cold-water-swimming for 14 consecutive day plus single i.p. injection of *Nepeta menthoides *(500 mg/kg)
Ctr + 100, n=6	warm-water swimming for 14 consecutive day plus single i.p. injection of *Nepeta menthoides *(100 mg/kg)
CWH + vehicle, n=6	cold-water-swimming for 14 consecutive day plus single i.p. injection of normal saline
Ctr + vehicle, n=6	warm-water swimming for 14 consecutive day plus single i.p. injection of normal saline
Intact rats, n=10	No intervention

*Water maze task*

Apparatus. The water maze was consisted of a pool (155 cm diameter) filled with water (23 ±1 °C) until 15 cm from the edge of the tank. A transparent Plexiglas platform (10 cm diameter) was located 1.5 cm below the water surface in the tank’s southeastern quadrant (target quadrant, Q1). The platform was the only escapable thing from the water. The walls surrounding the pool were decorated with distinct extra maze cues. These cues were kept in the fixed positions with respect to the swimming pool during the whole experiment to allow the animals finding the hidden platform.

Animal movements was recorded by a CCD camera (Panasonic Inc., Japan) hanging from the ceiling above the MWM apparatus and locomotion tracking was measured by the Ethovision software (version XT7, Netherland), a video tracking system for automated analyzing of animal’s behavior.

Protocol. The protocol was a modified version previously described by Frick *et al. *(11). In brief, learning step consisted of 60 s swimming for adaptation, and twelve trials, organized into three blocks of four trials separated by 30 min. Each animal was given a maximum of 60 s to reach to the platform, upon which it remained for 10 s. If the platform was not located within 60 s, the animal was placed on it by the experimenter. The next trial started immediately after removal from the platform. Escape latencies to find the hidden platform and swimming speeds were recorded in each trial for subsequent analysis.

Twenty four hours after last training trial, the spatial probe test was given. In spatial probe test, the platform was removed, and rats were allowed to swim for 60 s before they were removed. Animals were released in the water in a location that was exactly opposite from where the platform was placed. Behavior was recorded with the video tracking system and time spent in Q1was recorded.

After the probe trial, the platform was elevated above the water surface, covered by bright color aluminum foil, and placed in a different quadrant (northwestern quadrant). Rats were allowed to swim and find the visible platform during 60 s for four times in order to test their visual ability. All experiments were conducted between 10:00 and 15:00.

*Tissue preparation and biochemical analyses*

After behavioral tests, animals were sacrificed by cervical translocation, and the brains were removed from the skulls. The hippocampus tissue was separated from each hemisphere and was snapfrozen in liquid nitrogen and kept at −80 °C to time of biochemical analysis.

*Western blot analysis*

Western blot analysis was performed on hippocampi homogenates to determine Tau hyperphosphorylation levels. Briefly, the whole hippocampus was homogenized in four times the volume–weight of lysis buffer (Tris–HCl 50 mM, NaCl 150 mM, Triton X-100 0.1%, sodium deoxycholate 0.25%, SDS 0.1%, EDTA 1 mM, protease and phosphatase inhibitor) and the total protein extract was then obtained by centrifugation for 15 min at 12000 rpm at 4 °C. Protein concentration was determined by the Bradford assay, and equivalent amounts (60 μg) of each sample were subjected to SDS-PAGE electrophoresis. The proteins were transferred onto PVDF membranes (Millipore), for assessment of Phospho-Tau Ser 396, Total Tau, and β-actine, by using Bio-Rad immunoblotting apparatus. Afterward, the membranes were blocked in 5% nonfat dry milk in Tris-buffered saline containing 0.1% Tween 20 for 75 min at room temperature and then were incubated at 4 °C for 3 h with

Purified rabbit polyclonal anti-tau antibody PS396 (phosphor-Ser396; 1:800 v/v, Signalway Antibody), anti total tau (1:800 v/v, Signalway Antibody), and β-actin rabbit monoclonal antibody (1:1,000 v/v; cell signaling). The membranes were washed three times with 0.1% Tween 20 and TBS and then incubated with horseradish peroxidase conjugated gout anti- rabbit secondary antibodies (cell signaling) (1:10,000 v/v). The immunoreactive band signal intensity was visualized by enhanced chemiluminescence (ECL Plus, GE Healthcare Biosciences, Piscataway, NJ). The relative expression of the protein bands was quantified by scanning of the X-ray films and densitometric analysis with ImageJ software.

*Statistics*

All collected data were analyzed using the SPSS for Windows (Version 16). To compare first and third training blocks of each group, one-way repeated measures analysis of variance (ANOVA) followed by Bonferroni post hoc analysis was used. One-way ANOVA followed by LSD post hoc test was used to compare different experimental groups in each training trial and also to compare the time spent in target quadrant (Q1) as a measure of memory retrieval, by each group in the probe test. One-way ANOVA followed by LSD post hoc analysis was also used to compare the levels of phosphorylated tau between different experimental groups. A P<0.05 was considered significant. All error bars in figures are ± SEM (Standard Error of Mean).

## Results

*Spatial acquisition*

**Figure 1:**
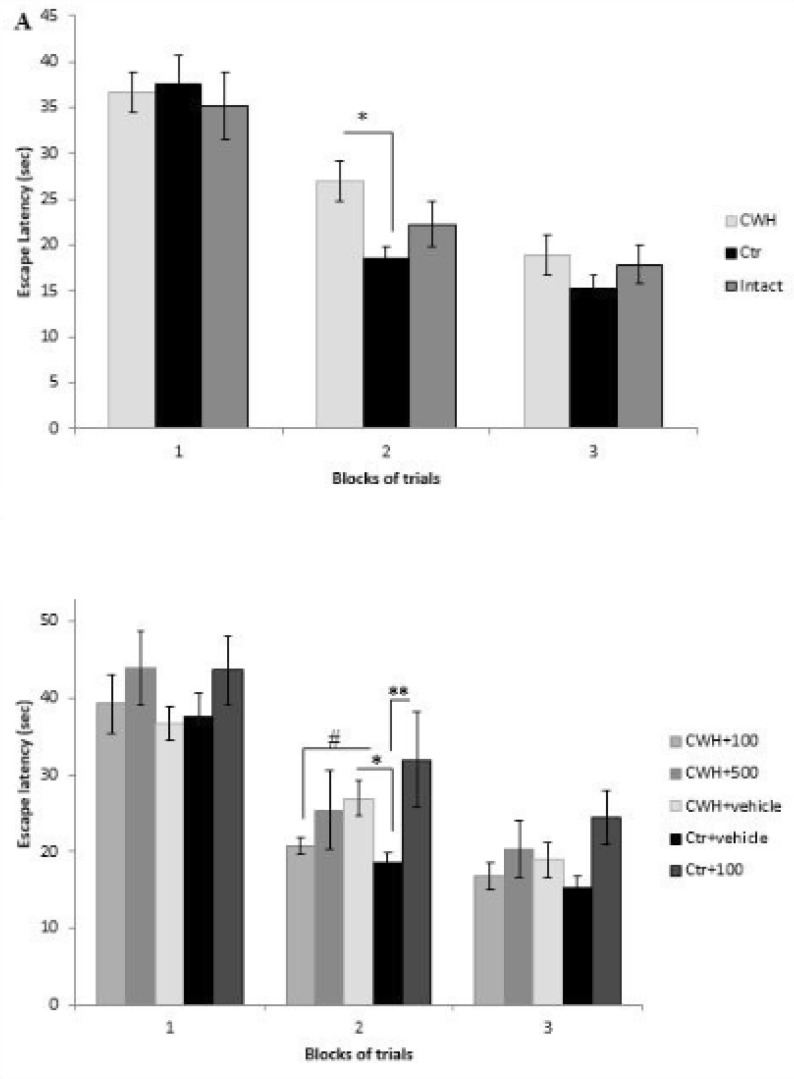
Learning procedure was impaired by chronic cold-water-induced hypothermia (CWH) and the impairment was neutralized by *i.p*. injection of *Nepeta menthoides*. A) Escape latencies (sec) of CWH, Ctr, and intact groups measured by Morris water maze. Hypothermia impairs learning in block 2 (*P=0.03). B) 100 mg/kg of the herb neutralized thelearning impairment of hypothermia but the extract in CWH+500 and Ctr+100 groups had negative activity. Data are means ± SEM

In the acquisition phase of the MWM, statistical analysis (one-way repeated measures ANOVA) showed that all animals in the experimental groups can find the hidden platform in a significant lesser time in the third training trial in comparison with the first trial (P < 0.001). However, comparing CWH with its control (Ctr group) revealed that chronic hypothermia has negative effect on block 2 of the training trials (P=0.03, one-way ANOVA). Furthermore, to show that forced swimming stress per se has no deleterious effect on spatial learning we compared the Ctr group with a group of intact animals and showed that there is no significant difference between control and intact rats (Figure 1 A).

To clarify whether *Nepeta menthoides *can reverse learning impairment of hypothermia, 100 and 500 mg/Kg of the herb extract were injected 24 hours before the training trials. The results showed that, in comparison with their appropriate controls, the dose of 100 mg/Kg – but not 500 mg/Kg- can ameliorate learning impairment in hypothermic rats (Figure 1 B). Surprisingly, 500 mg/Kg of the herb neutralized the positive effects of the dose 100 mg/Kg on learning. Furthermore, learning capacity of rats in Ctr+100 group was also disturbed (*P*Ctr+vehicleVs Ctr+100 =0.01). It means that lower dose of this “hot” herb can neutralize the destructive effects of cold water on learning but a higher dose in hypothermic rats or the same dose in normothermic ones abolish learning.

*Probe test*

**Figure 2:**
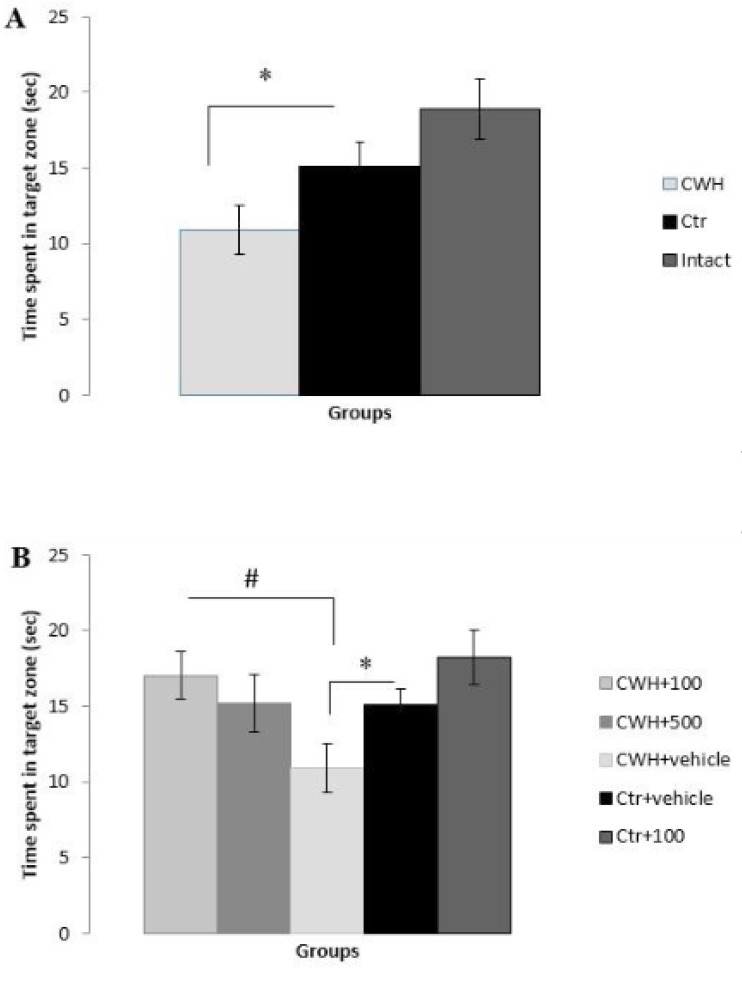
Spatial memory retrieval based on time spent in target zone (sec). A) Cold-water- induced hypothermia (CWH) impaired memory (*P<0.05) while forced swimming stress had no destructive effect on learning. B) *i.p*. injection of 100 mg/Kg of the herb reversed cold-water-induced memory impairment (#P<0.05). Data are means ± SEM

Results of the probe trial as measured by the time spent in Q1 indicated memory impairment in hypothermic rats (Figure 2A). In other words, rats exposed to 14-day CWH spent lesser amounts of time in Q1 (P < 0.05), and therefore showed impaired memory retrieval versus the control group. This is while 14 days of warm water swimming stress had no deleterious effect on spatial memory in comparison with intact rats (Figure 2A). The results also showed that

*Nepeta menthoides *in the dose of 100 mg/Kg reverse memory impairment of hypothermic rats (P < 0.05) (Figure 2B) while the effect of the dose 500 mg/Kg is not significant. 

Swimming speed decreased across blocks of acquisition but there were no statistical differences between groups indicating no motor disturbance. Swimming speed of rats was also similar in probe trial. In the visible test taken after probe test, all groups could find platform indicating no visual impairment in the animals.

*Western blotting*

**Figure 3:**
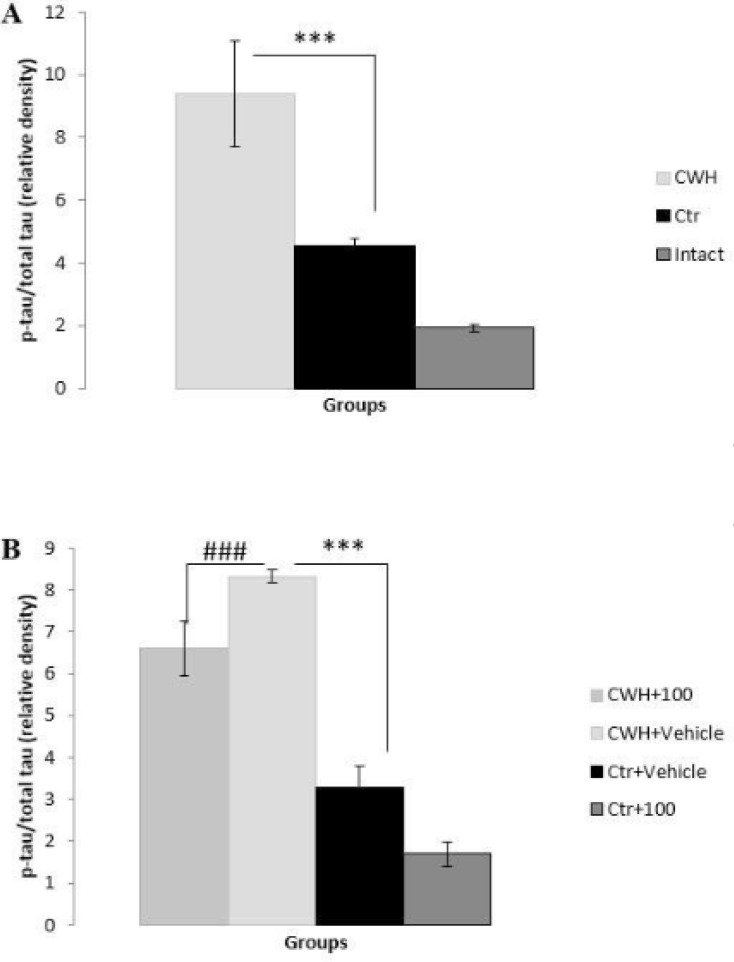
Levels of tau hyperphosphorylation measured by western blotting. A) Cold-water- induced hypothermia induced tau hyperphosphorylation (***p<0.001). B) *i.p*. injection of the herb reduced the level of tau hyperphosphorylation (###p<0.001). Data are means ± SEM

To clarify the probable mechanisms through which hypothermia impairs memory, phosphorylation of tau protein was determined quantitatively by Western blot analysis. The results showed a significant increase in phosphorylation of tau protein at Ser396 epitope after 14 days of CWH (P < 0.001) (Figure 3A). Tau hyperphosphorylation also increased in Ctr group but this increase was much less than CWH group. The results also indicated that 100 mg/Kg of *Nepeta menthoides *in CWH+100 and Ctr+100 groups reduce tau hyperphosphorylation (Figure 3B). The reduction in CWH+100, however, was not strong enough to completely neutralize the tau hyperphosphorylation which was induced by hypothermia. 

*Tissue preparation and biochemical analyses*

After behavioral tests, animals were sacrificed by cervical translocation, and the brains were removed from the skulls. The hippocampus tissue was separated from each hemisphere and was snapfrozen in liquid nitrogen and kept at −80 °C to time of biochemical analysis. 

*Western blot analysis*

Western blot analysis was performed on hippocampi homogenates to determine Tau hyperphosphorylation levels. Briefly, the whole hippocampus was homogenized in four time the volume–weight of lysis buffer (Tris–HCl 50 mM, NaCl 150 mM, Triton X-100 0.1%, sodium deoxycholate 0.25%, SDS 0.1%, EDTA 1 mM, protease and phosphatase inhibitor) and the total protein extract was then obtained by centrifugation for 15 min at 12000 rpm at 

4 °C. Protein concentration was determined by the Bradford assay, and equivalent amounts (60 μg) of each sample were subjected to SDS-PAGE electrophoresis. The proteins were transferred onto PVDF membranes (Millipore), for assessment of Phospho-Tau Ser 396, Total Tau, and β-actine, by using Bio- Rad immunoblotting apparatus. Afterward, the membranes were blocked in 5% nonfat dry milk in Tris-buffered saline containing 0.1% Tween 20 for 75 min at room temperature and then were incubated at 4 °C for 3 h with 

Purified rabbit polyclonal anti-tau antibody PS396 (phosphor-Ser396; 1:800 v/v, Signalway Antibody), anti total tau (1:800 v/v, Signalway Antibody), and β-actin rabbit monoclonal antibody (1:1,000 v/v; cell signaling). The membranes were washed three times with 0.1% Tween 20 and TBS and then incubated with horseradish peroxidase conjugated gout anti- rabbit secondary antibodies (cell signaling) (1:10,000 v/v). The immunoreactive band signal intensity was visualized by enhanced chemiluminescence (ECL Plus, GE Healthcare Biosciences, Piscataway, NJ). The relative expression of the protein bands was quantified by scanning of the X-ray films and densitometric analysis with ImageJ software.

## Discussion

This study shows the destructive effect of chronic cold-water-induced hypothermia on learning and memory. CWH consists of physical (coldness of water) and psychological (forced swimming) stressors (12). In the present study, forced swimming in warm water (32 °C) was utilized as control group to separate the psychological stressor from the physical one. As the results show, while cold-water swimming for 14 consecutive days impairs memory retrieval, swimming in warm water has no negative effect on this behavioral parameter. Therefore, it is concluded that memory impairment is a result of chronic hypothermia, not of swimming stress.

Cold-water swimming was utilized in this study to investigate the role of hypothermia, especially that of brain hypothermia, on learning and memory. Previously, Moser and colleagues in 1993 and Taltavull and colleagues in 2003 indicated that cold-water swimming considerably decreases the brain temperature (9, 10). ITM presents coldness of brain (also called brain cold intemperament) as the main cause of dementia (2, 3). Therefore, this study tried to examine the effect of brain coldness on learning and memory. According to our results, chronic hypothermia can impair learning and memory, and one of the probable mechanisms of this impairment is tau hyperphosphorylation.

Tau hyperphosphorylation is one of the two hallmarks of AD. Tau protein is a microtubule- associated protein and one of its main functions is to modulate the stability of axonal microtubules (13).When hyperphosphorylation occurs, tau not only loses its function to assemble tubulins into microtubules, but instead inhibits assemblage and disrupts microtubules (13), which results in neuronal death. There is a relationship between the sites of hyperphosphorylation and the severity of AD. One of the epitopes which is hyperphosphorylated in advanced stages of AD is Ser 396 (14); in this study, we showed that chronic hypothermia induces tau hyperphosphorylation in this epitope. Concurrency of memory impairment and tau hyperphosphorylation after 14 consecutive days of hypothermia indicates that there is a similarity between memory impairment induced by hypothermia with Alzheimer’s dementia.

Another goal of this study was to examine the effects of *Nepeta menthoides *as a traditional “hot” herb in neutralization of hypothermia-induced learning and memory impairment. The results showed that lower dose of this herb reverses learning and memory impairment, but higher dose has negative effects on learning. These findings are in accordance with ITM notifications. ITM prescribes “hot” herbs for “cold” diseases to restore the equilibrium to the unhealthy organs. If overspending in prescription of hot herbs happen, the drugs can produce “hot” diseases instead of treating “cold” ones. In ITM’s words, overdose of *Nepeta menthoides *produces learning impairment because of “hot intemperament” of the brain.

The dose 100 mg/Kg of the herb reduced hyperphosphorylation of tau protein. Thus, one of the probable anti-dementia mechanisms of *Nepeta menthoides *is to prevent tau hyperphosphorylation. More investigation on anti-AD activities of this herb is recommended. 

## Conclusion

As a conclusion, regarding the ITM theory about the role of brain coldness on dementia, we try to examine the effect of brain hypothermia on learning and memory. Our findings showed that chronic hypothermia can impair learning capacity as well as memory retrieval. This memory impairment accompanied with tau hyperphosphorylation; thus, it can be considered similar to AD dementia. A traditional “hot” herb, *Nepeta menthoides*, neutralized the memory impairment induced by hypothermia. Reduction in tau hyperphosphorylation level was one of the mechanisms of its anti-dementia activities. Based on our findings, the traditional “hot” herbs would be good candidates for more investigation on AD drug therapy.
